# Effect of sialyllactose on growth performance and intestinal epithelium functions in weaned pigs challenged by enterotoxigenic *Escherichia Coli*

**DOI:** 10.1186/s40104-022-00673-8

**Published:** 2022-03-03

**Authors:** Qiming Duan, Daiwen Chen, Bing Yu, Zhiqing Huang, Yuheng Luo, Ping Zheng, Xiangbing Mao, Jie Yu, Junqiu Luo, Hui Yan, Jun He

**Affiliations:** 1 Institute of Animal Nutrition, Sichuan Agricultural University, Sichuan Province Chengdu, 611130 People’s Republic of China; 2 Key Laboratory of Animal Disease-resistant Nutrition, Sichuan Province Chengdu, 611130 People’s Republic of China

**Keywords:** Immunity, Inflammation, Intestinal epithelium, Sialyllactose, Weaned pigs

## Abstract

**Background:**

Sialyllactose (SL) is one of the most abundant oligosaccharides present in porcine breast milk. However, little is known about its effect on growth performance and intestinal health in weaned pigs. This study was conducted to explore the protective effect of SL on intestinal epithelium in weaned pigs upon enterotoxigenic *Escherichia coli* (ETEC) challenge.

**Methods:**

Thirty-two pigs were randomly divided into four treatments. Pigs fed with a basal diet or basal diet containing SL (5.0 g/kg) were orally infused with ETEC or culture medium.

**Results:**

SL supplementation elevated the average daily gain (ADG) and feed efficiency in the ETEC-challenged pigs (*P* < 0.05). SL also improved the digestibilities of dry matter (DM), gross energy (GE), and ash in non-challenged pigs (*P* < 0.05). Moreover, SL not only elevated serum concentrations of immunoglobulins (IgA, IgG, and IgM), but also significantly decreased the serum concentrations of inflammatory cytokines (TNF-α, IL-1β, and IL-6) upon ETEC challenge (*P* < 0.05). Interestingly, SL increased the villus height, the ratio of villus height to crypt depth (V:C), and the activities of mucosal sucrase and maltase in the jejunum and ileum (*P* < 0.05). SL also elevated the concentrations of microbial metabolites (e.g. acetic acid, propanoic acid, and butyric acid) and the abundance of *Lactobacillus*, *Bifidobacterium*, and *Bacillus* in the cecum (*P* < 0.05). Importantly, SL significantly elevated the expression levels of jejunal zonula occludins-1 (ZO-1), occluding, and fatty acid transport protein-4 (FATP4) in the ETEC-challenged pigs (*P* < 0.05).

**Conclusions:**

SL can alleviate inflammation and intestinal injury in weaned pigs upon ETEC challenge, which was associated with suppressed secretion of inflammatory cytokines and elevated serum immunoglobulins, as well as improved intestinal epithelium functions and microbiota.

**Supplementary Information:**

The online version contains supplementary material available at 10.1186/s40104-022-00673-8.

## Background

Weaning is one of the most stressful events for neonatal pigs, as weaning deprives their protections from maternal passive immunity and may contribute to intestinal and immune system dysfunctions that lead to reduced growth and feed intake, particularly during the first week after weaning [1]. Post weaning diarrhea (PWD) is a common problem in pig production, and enterotoxigenic *Escherichia coli* (ETEC) has been considered to be the main bacterial cause of PWD and intestinal injury (accounts for more than 45% of piglet diarrhea). Previous study indicated that ETEC can adhere to intestinal epithelial cells and cause intestinal injury through endotoxin and exotoxin [2, 3]. In past decades, antibiotics have been widely used as therapeutic drugs for ETEC infection to prevent diarrhea and intestinal injury in pig production [4]. However, the abuse of antibiotics can lead to bacterial resistance and drug residues in animal products [5]. Therefore, developing of substitutes for traditionally used antibiotics has attracted considerable research interest worldwide.

Breast milk has been considered to be the most ideal food for neonatal animals including pigs, as it contains nearly all necessary nutrients for neonatal animals [6]. Moreover, the breast milk contains a variety of bioactive substances such as antibacterial peptides, growth factors, and oligosaccharides [7]. Milk oligosaccharides (MOs) are an important component of mammalian milk carbohydrate, and are the third largest component in breast milk [8]. Currently, increasing evidence is generated for specific functions of specific MOs, being anti-adhesive, immune-modulating, or a targeted prebiotic for specific desirable bacterial/microbiota strains in early infant development [9]. MOs are composed of the five monosaccharides glucose (Glc), galactose (Gal), N-acetylglucosamine (GlcNAc), fucose (Fuc) and sialic acid (Sia). Sialyllactose (SL), which is the most abundant components of milk oligosaccharides [10] and is a compound where the N-acetyl-D-neuraminic acid (Neu5Ac or SA) unit is connected to the galactose unit of lactose and essentially it is a sialic acid bound to the lactose molecule [11]. Previous studies indicate that MOs can improve baby’s health and development by promoting the growth of intestinal beneficial microflora (e.g. *Bifidobacterium* and *Bacillus*) [12], serving as anti-adhesive antimicrobials to directly reduce microbial infections [13], and providing sialic acid to help brain development [14]. Moreover, MOs can also be used to protect piglets against rotavirus infection and shorten the duration of diarrhea [15].

Although, there are many studies on milk oligosaccharides, the effect of SL on growth performance and intestinal epithelial functions in weaned pigs is just beginning to be explored. This study was conducted to explore whether SL supplementation could attenuate intestinal inflammation and epithelium injury in weaned pigs induced by ETEC. This study will also provide convincing evidence on the beneficial effect of milk oligosaccharides and offer key insights into its potential mechanisms of action.

## Materials and methods

### Bacterial strains and culture

Pathogenic *Escherichia coli* (ETEC) was purchased from China veterinary culture collection center (CVCC, Beijing, China). The serotype of ETEC was o149: K91, k88ac, and the strain number of CVCC was 225. It was a vacuum freeze-dried strain. The ETEC with strong toxicity was produced through resuscitation and proliferation culture in our laboratory. Luria-Bertani (LB) and LB agar broth were prepared (Yeast Extract*,* 0.5 g*;* NaCl, 1 g; Peptone, 1 g; double-distilled water, 50 mL; agar powder, 2% for LB agar broth) and sterilized under 121 °C, 0.11 MPa for 20 min (pH = 6). The bacteria were resuscitated in 3 mL of Luria Bertani (LB) broth at 37 °C with shaking for 24 h and inoculated on LB agar. A single colony was inoculated into 50 mL of LB broth, cultured overnight at 37 °C and 250 rpm, then subcultured and serially diluted on LB agar for bacterial counts [16].

### Experimental design and diet

A total of 32 male Duroc × Landrace × Yorkshire pigs weaned at 21 days of age (with an average body weight of 6.66 ± 0.14 kg) were selected and randomly allotted into a 2 (SL) × 2 (ETEC) factorial experiment of four treatments composed of CON (pigs were fed with a basal diet), CSL (pigs were fed with basal diet containing 5 g/kg SL product), ECON (pigs were fed with a basal diet and challenged by ETEC), ESL (pigs were fed with basal diet containing 5 g/kg SL product and challenged by ETEC). Sialylactose (SL, ≥ 95%) was obtained from Glycom A/S. SL is produced via microbial fermentation and is a trisaccharide made from glucose, galactose and N-acetylneuraminic acid. The concentration of SL in the diet is 5 g/kg, which was decided according to the oligosaccharides content in the mature milk of sow’s (5 to 10 g/L) and previous study on piglets [17]. Pigs were received the same parental nutrition and management (e.g. sows were fed with the same diet, synchronization estrous). The trial last for 28 d. On d 26, the challenge groups were orally treated with 100 mL of LB culture containing 1×10^10^ CFU/mL of ETEC by using an orogastric tube last for 3 d, whereas the non-challenge groups were orally treated with equivalent amount of culture medium [18]. The basal diet (Table 1) was formulated to meet the swine nutrient requirements recommended by National Research Council (NRC, 2012) [19]. Pigs were individually housed in 1.5 × 0.7 m^2^ metabolism cage and were given ad libitum access to feed and fresh water with the room temperature controlled between 25 and 28 °C, relative humidity 65% ± 5%.

**Table 1:** Experiment basal diet composition and nutrient level

Ingredients	%	Nutrient level	contents
Corn	28.31	Digestible energy, calculated, MJ/kg	14.78
Extruded corn	24.87	Crude Protein, %	19.68
Soybean meal	8.50	Calcium, %	0.81
Extruded full-fat soybean	10.30	Available phosphorus, %	0.55
Fish meal	4.20	Lysine, %	1.35
Whey powder	7.00	Methionine, %	0.42
Soybean protein concentrate	8.00	Methionine + cysteine, %	0.60
Soybean oil	2.00	Threonine, %	0.79
Sucrose	4.00	Tryptophan, %	0.22
Limestone	0.90		
Dicalcium phosphate	0.50		
NaCl	0.30		
L-Lysine HCl, 78%	0.47		
DL-Methionine	0.15		
L-Threonine, 98.5%	0.13		
Tryptophan, 98%	0.03		
Chloride choline	0.10		
Vitamin premix ^1^	0.04		
Mineral premix ^2^	0.20		
Total	100		

^1^The vitamin premix provided the following per kg of diet: 9000 IU of VA, 3000 IU of VD 3, 20 IU of VE, 3 mg of VK_3_, 1.5 mg of VB_1_, 4 mg of VB_2_, 3 mg of VB_6_, 0.02 mg of VB_12_, 30 mg of niacin, 15 mg of pantothenic acid, 0.75 mg of folic acid, and 0.1 mg of biotin. ^2^ The mineral premix provided the following per kg of diet: 100 mg Fe, 6 mg Cu, 100 mg Zn, 4 mg Mn, 0.30 mg I, 0.3 mg Se

^2^The diet was formulated based on the recommendation of NRC (2012)

### Sample collection

Feed samples were collected at the beginning of the experiment and stored at − 20 °C for nutrient analysis, during the d 22–25 of the experiment, fresh fecal samples were collected immediately after excretion from pigs in each cage, weighed, and adding 10 mL 10% H_2_SO_4_ solution to per 100 g of fresh fecal. The feed and fecal samples were dried at 65 °C for 2 d, and ground to pass through a 1-mesh screen, and then stored at − 20 °C until measurement for nutrients digestibility. Blood samples were obtained on d 22 by jugular vein puncture and placed in two 10-mL vacuum tubes. One for blood parameters analyzed and the serum was collected after centrifugation of another tube at 3500 × *g* and stored at − 20 °C until the serum indexes analysis. Pigs were anesthetized by intravenous injection with sodium pentobarbital (200 mg/kg BW), and the tissues of the duodenum, jejunum and ileum were immediately isolated at the end of the trial. At the same time, the duodenum, jejunum and middle ileum (4 cm from the middle of each intestine) were gently rinsed with cold phosphate buffered saline (PBS), and then fixed in 4% paraformaldehyde solution for morphological analysis. In addition, the cecal digestive products were collected, and the intestinal mucosa was obtained from the residual intestinal segments with a scalpel blade and placed in a freezing tube, then frozen by immersion in liquid nitrogen, and stored at − 80 °C until analysis.

### Apparent total tract nutrient digestibility analysis

The frozen dried and milled feed and fecal samples were used for nutrient digestibility analysis, which uses acid insoluble ash (AIA) as endogenous indicators. The dry matter (DM), crude protein (CP), ether extract (EE) and ash contents were determined according to AOAC [20], whereas the GE content was measured by an adiabatic bomb calorimeter (LECO, St. Joseph, Michigan, USA) to calculate the apparent total tract digestibility. All contents were calculated by following formula: (100-A1F2/A2F1 × 100) [21] A1: digesta nutrient; A2: digesta AIA; F1: diet AIA; F2: digesta AIA.

### Serum proinflammatory cytokines and immunoglobulin detection

The concentration of proinflammatory cytokines (TNF-α, IL-1β and IL-6) and immunoglobulin (IgG, IgM, and IgA) in serum were determined followed by the instructions of a commercial available porcine Enzyme-Linked Immunosorbent Assay (ELISA) kits (Shanghai Meimian Biotechnology Co., Ltd., Shanghai, China). All procedures were guided by manuals of the kits. For quantification, the standards provided in the kits were used to generate standard curves.

### Histomorphology analysis of intestinal segment

The fixed with 4% paraformaldehyde intestinal segments were dehydrated through a graded series of ethanol and then embedded in paraffin [21]. Cross-sections of each sample were prepared, stained with hematoxylin and eosin (H&E), and then sealed with neutral resin. Finally, ultrathin sections of the duodenal, jejunal and ileal samples were examined for crypt depth and villus height with image processing and analysis system (Image-Pro Plus 6.0). The method of measurement of crypt depth and villus height was followed by King’s and Wan’s. A total of 10 intact, well-oriented, crypt-villus units were analyzed in triplicate per intestinal segment [22, 23]. Calculate the ratio of villus height to crypt depth (V:C) from the above data.

### Enzyme activity

The frozen duodenal, jejunal and ileal mucosa were homogenized in chilled saline at a ratio of 1:9 (w/v) for 15 min. The homogenate was centrifuged at 3500 × *g* for 10 min at 4 °C and the supernatant was used to determine the enzyme activities. The activity of alkaline phosphatase (AKP) was determined using the alkaline phosphatase assay kit (A059–2-2) purchased from Nanjing Jiancheng Biotechnology Co., Ltd. (Nanjing, China). The enzyme-linked immunosorbent assay (ELISA) kits purchased from Shanghai Enzyme-linked Biotechnology Co., Ltd. (Shanghai, China) were used for detections of lactase (Porcine Lactase ELISA Kit ml712060), sucrase (Porcine Sucrase ELISA Kit ml712026), and maltase (Porcine Maltase ELISA Kit ml712030). All procedures were carried out according to the instructions of the kits. The absorbance of each reaction was determined using the spectrophotometer (UV–VIS Spectrophotometer, Leng Guang SFZ1606017568, Shanghai, China.) at a wavelength of 450 nm. Enzyme activity was defined as hydrolysis of 1 mol of the substrate per mg of protein tissue per minute under the condition of 37 °C, pH = 6.0.

### Caecal microbiological analysis

Approximately 200 mg caecal digesta was weighed and treated using the Stool DNA Kits (Omega Bio-Tek, Doraville, CA, USA) following the manufacturer’s instruction to extract total DNA for quantification real-time PCR, which was performed by conventional PCR on the Opticon DNA Engine (Bio-Rad). Total bacteria were detected by the reaction which runs in a volume of 25 μL with 1 μL of forward and 1 μL of reverse primers (100 nmol/L), 12.5 μL SYBR Premix Ex Taq (2 × concentrated), 2 μL template DNA, 1 μL 50 × ROX Reference Dye*3 and 7.5 μL of RNase-Free ddH_2_O. The SuperReal PreMix (Probe) kit (Tiangen Biotech Co., Ltd., Beijing, China) was used for *Lactobacillus*, *E. coli*, *Bacillus* and *Bifidobacterium* detection and primers and fluorescent oligonucleotide probes presented in Table S1. Each reaction was run in a volume of 25 μL with 12.5 μL 2 × Super Real PreMix (Probe), 1 μL of forward and 1 μL of reverse primers (100 nmol/L), 1 μL 50 × ROX Reference Dye*3, 1 μL probe (100 nmol/L), 2 μL DNA and 6.5 μL of RNase-Free ddH_2_O. All reaction protocol was composed of one cycle of pre-denaturation at 95 °C for 15 min; forty cycles of denaturation at 95 °C for 3 s; annealing and extension at 60 °C for 30 s. The Cycle threshold (Ct) values and baseline settings were determined by automatic analysis settings, and the copy numbers of the target group for each reaction were calculated from the standard curves, which were generated by constructing standard plasmids by a 10-fold serial dilution of plasmid DNA (1 × 10^1^ to 1 × 10^9^ copies/μL).

### Metabolite concentrations in cecal digesta

The SCFA (acetic acid, propionic acid, and butyric acid) concentrations were determined using a gas chromatograph system (VARIAN CP-3800, Varian, Palo Alto. CA, USA; capillary column 30 m × 0.32 mm × 0.25 μm film thickness) following previous method [24]. After vortex, the digesta was centrifuged at 4 °C for 10 min (12,000 × *g*), and the supernatant (1 mL) was then transferred into an Eppendorf tube (2 mL) and mixed with 0.2 mL metaphosphoric acid. After 30 min incubation at 4 °C, the tubes were centrifuged at 4 °C for 10 min (12,000 × *g*) and aliquots of the supernatant (1 μL) were analyzed using the GC with a flame ionization detector and an oven temperature of 100–150 °C. The polyethylene glycol column was operated with highly purified N2 as the carrier gas at 1.8 mL/min.

### Isolation and reverse transcription of RNA and q-PCR

The frozen duodenal, jejunal and ileal mucosa samples (about 0.1 g) were ground in liquid nitrogen and homogenized in 1 mL of RNAiso Plus (Takara Biotechnology Co., Ltd., Dalian, China) to extract total RNA followed the manufacturer’s instructions, and the purity and concentration of total RNA were detected by using a spectrophotometer (NanoDrop 2000, Thermo Fisher Scientific, Inc., Waltham, MA, USA), samples which OD260/OD280 ratio ranged from 1.8 to 2.0 were deemed appropriate. Subsequently, a volume equivalent to 1 μg total RNA from each duodenal, jejunal, and ileal sample was used for reverse transcription into cDNA, which based on the protocol of PrimeScript™ RT reagent kit with gDNA Eraser (Takara Biotechnology Co., Ltd., Dalian, China). This process consists of two steps: I: 37 °C for 15 min, II: 85 °C for 5 s.

The expression level of the target gene in intestinal mucosa was quantified using q-PCR, the oligonucleotide primers sequences used in q-PCR were presented in Table S1, qPCR was performed with the SYBR® Green PCR I PCR reagents (Takara Bio Inc., Dalian, China) using a CFX96 Real-Time PCR Detection System (Bio-Rad Laboratories, Hercules, CA, USA). All cDNA samples were detected in triplicate. The reaction mixture (10 μL) contained 5 μL SYBR Premix Ex Taq II (Tli RNaseH Plus), 0.5 μL forward primer, 0.5 μL reverse primer, 1 μL cDNA and 3 μL RNase-Free water. The protocol used in q-PCR was as followed: 95 °C for 30 s, followed by 40 cycles: at 95 °C for 5 s and 60 °C for 34 s. The generated Gene-specific amplification products were confirmed by melting curve analysis after each real-time quantitative PCR assay. The housekeeping gene β-actin was used to standardize the mRNA expression level of target genes, which calculated based on the 2^–ΔΔCt^ method [25].

### Sample size calculation and statistical analysis

The minimal sample size was calculated based on the experimental design (repeated-measures, between factors ANOVA) determining the SL × ETEC effect to the intestinal health as the primary outcome measure. We used G*Power software (version 3.1.9.2) for the power analysis with following variables; the power = 0.8, significant level = 0.05, and effect size = 0.35 in the experiment. The effect size was estimated based on the results from a preliminary study. Hence, the required minimal sample size was five pigs per group.

The data collected before the ETEC challenge was analyzed by one-way ANOVA. After the challenge, the data were analyzed by two-way ANOVA with the General Linear Model (GLM) procedure of SPSS as a 2 (SL) × 2 (ETEC) factorial design. *P*-value < 0.05 was deemed to be significant and the *P*-value between 0.05 and 0.1 to show a significant trend. Duncan’s multiple range test was used based on the analysis of ANOVA, which showed a significant difference. All data were analyzed by SPSS 24.0 (IBM, Chicago, IL, USA) and GraphPad (version 9) software (GraphPad Software Inc., CA, USA). Results are expressed as means with their standard errors.

## Results

### Effect of SL on growth performance and nutrients digestibility in weaned pigs upon ETEC challenge

As shown in Table 2, ETEC challenge decreased ADG and increased F:G in the weaned pigs (*P* < 0.05). Moreover, we observed a trend toward an interaction effect of SL and ETEC, when this trend toward an interaction effect was investigated with post-hoc test, we found that while the ETEC increased the F:G, this effect was prevented by the SL treatment. SL increased the digestibilities of DM, Ash, and GE in non-challenged pigs (*P* < 0.05). The digestibility of Ash was also higher in the ESL group than in the ECON group (*P* < 0.05) (Table 3).

**Table 2:** Effect of SL supplementation on growth performance in weaned pigs upon ETEC challenge

Items	Treatments				SEM	*P*-value		
	CON	CSL	ECON	ESL		SL	ETEC	Interaction
1−19 d								
ADFI, g/d	420.50	425.06	418.43	425.59	19.07			
ADG, g/d	281.20	294.60	285.12	292.48	13.98			
F:G	1.50	1.44	1.47	1.46	0.04			
19−21 d								
ADFI, g	618.50^ab^	705.43^a^	527.86^b^	590.90^ab^	26.37	0.14	0.05	0.81
ADG, g	510.53^a^	633.33^a^	361.33^b^	516.00^a^	31.14	0.01	0.01	0.74
F:G	1.21^b^	1.11^b^	1.46^a^	1.15^b^	0.09	0.02	0.08	0.08

*ADFI* average daily feed intake, *ADG* average daily gain, *F:G* Feed:Gain ratio

^1^Mean and total SEM are list in Separate columns, *n* = 8

^2^a, b, c mean values within a row with unlike superscript letters were significantly different, *P* < 0.05

^3^*CON*, pigs were fed with a basal diet; *CSL*, pigs were fed with a SL containing diet, 5 g/kg; *ECON*, pigs were fed with a basal diet and challenged by ETEC; *ESL*, pigs were fed with a SL containing diet and challenged by ETEC

**Table 3:** Effect of SL supplementation on nutrients digestibility in weaned pigs

Items	Treatments				SEM	*P*-value
	CON	CSL	ECON	ESL		
DM, %	88.86^b^	91.05^a^	89.09^b^	90.17^ab^	0.31	<0.01
CP, %	86.87	89.36	86.56	87.34	0.55	0.15
EE, %	66.05	72.24	66.73	71.46	1.40	<0.01
Ash, %	85.89^b^	89.76^a^	86.59^b^	89.85^a^	0.61	<0.01
GE, %	89.45^b^	91.31^a^	89.13^b^	90.62^ab^	0.38	0.02

*DM* dry matter, *CP* crude protein, *EE* ether extract

^1^Mean and total SEM are list in Separate columns, *n* = 8

^2^a, b mean values within a row with unlike superscript letters were significantly different, *P* < 0.05

^3^*CON*, pigs were fed with a basal diet; *CSL*, pigs were fed with a SL containing diet, 5 g/kg; *ECON*, pigs were fed with a basal diet and challenged by ETEC; *ESL*, pigs were fed with a SL containing diet and challenged by ETEC

### Effect of SL on serum immunogloblins and inflammatory cytokines in weaned pigs upon ETEC challenge

**Fig. 1:**
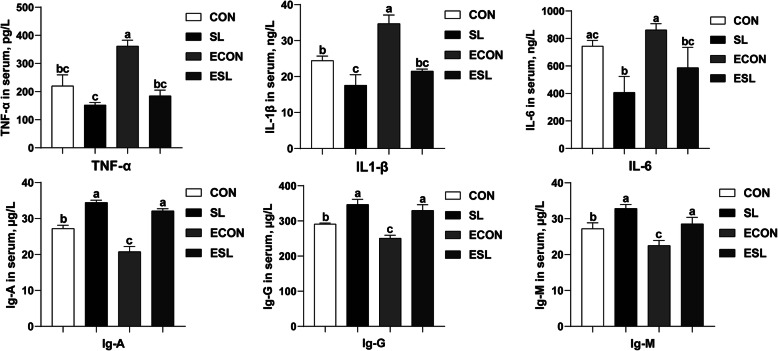
Effect of SL supplementation on serum concentrations of inflammatory cytokines and immunoglobulins. TNF-α, tumor necrosis factor-α; IL-1β, interleukin1-β; IL-6, interleukin-6; IgA, immunoglobulins A; IgG, immunoglobulins G; IgM, immunoglobulins M. a, b, c mean values within a row with unlike superscript letters were significantly different (*P* < 0.05). CON, pigs were fed with a basal diet; CSL, pigs were fed with a SL containing diet, 5 g/kg; ECON, pigs were fed with a basal diet and challenged by ETEC; ESL, pigs were fed with a SL containing diet and challenged by ETEC

As shown in Fig. 1, ETEC challenge significantly decreased the serum concentrations of IgA, IgG, and IgM (*P* < 0.05). However, SL supplementation increased their concentrations in the non-challenged pigs (*P* < 0.05). A trend toward an interaction effect of SL and ETEC was observed that the decreasing of IgA upon ETEC challenged was prevented by the SL treatment. The serum inflammatory cytokines such as the TNF-α and IL-1β were higher in the ECON group than in the CON group (*P* < 0.05). However, SL supplementation significantly decreased their concentrations in the ETEC-challenged pigs (*P* < 0.05) and increased the IL-6 concentration in non-challenged pigs.

### Effect of SL supplementation on intestinal morphology and mucosal enzyme activity in weaned pigs upon ETEC challenge

**Fig. 2:**
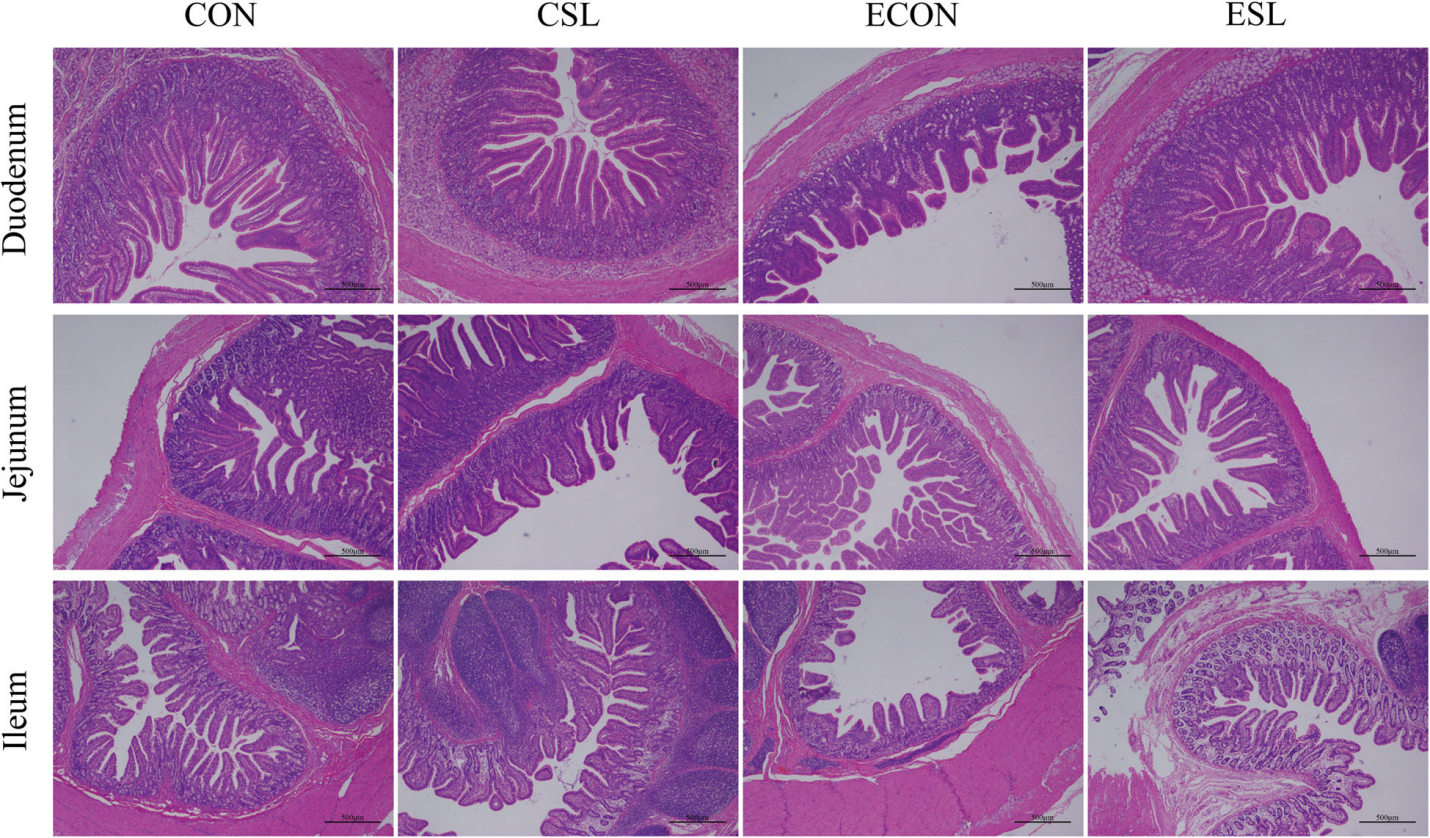
Effect of SL supplementation on intestinal morphology in weaned pigs upon ETEC challenge (H&E; × 40). CON, pigs were fed with a basal diet; CSL, pigs were fed with a SL containing diet, 5 g/kg; ESL, pigs were fed with a basal diet and challenged by ETEC; ESL, pigs were fed with a SL containing diet and challenged by ETEC

As shown in Table 4 and Fig. 2, ETEC challenge decreased the villus height in the jejunum and ileum (*P* < 0.05). However, SL supplementation significantly increased the jejunal villus height in ETEC-challenged pigs (*P* < 0.05). For the V:C, ETEC challenge significantly decreased ratio of V:C in the duodenum, jejunum, and ileum (*P* < 0.05). Moreover, two trends toward an interaction effect of SL and ETEC in ileum were observed, when these trends toward an interaction effect were investigated with post-hoc test, we observed that while the ETEC increased the crypt depth and decreased V:C, these effects were prevented by the SL treatment (*P* < 0.05). As shown in Table 5, ETEC challenge decreased the activities of maltase and lactase in the duodenal and ileal mucosa, respectively. However, SL supplementation significantly elevated their activities in the non-challenged pigs (*P* < 0.05). Moreover, the sucrase activity in the duodenal, jejunal and ileal mucosa of the CSL group was significantly higher than that in the CON group (*P* < 0.05).

**Table 4:** Effect of SL supplementation on intestinal morphology in weaned pigs upon ETEC challenge

Items	Treatments				SEM	*P*-value		
	CON	CSL	ECON	ESL		SL	ETEC	Interaction
Duodenum								
Villus height, μm	439.83^b^	573.03^a^	367.14^b^	393.24^b^	25.47	0.07	0.01	0.21
Crypt depth, μm	153.13	152.28	169.47	153.70	5.66	0.49	0.46	0.53
V:C	2.87^b^	3.76^a^	2.17^c^	2.56^bc^	0.16	0.01	0.00	0.18
Jejunum								
Villus height, μm	397.09^a^	407.55^a^	252.71^b^	385.47^a^	17.19	<0.01	<0.01	<0.01
Crypt depth, μm	130.50^ab^	100.38^b^	155.96^a^	102.66^b^	6.35	<0.01	0.17	0.24
V:C	3.04^b^	4.06^a^	1.62^c^	3.75^ab^	0.28	<0.01	0.01	0.14
Ileum								
Villus height, μm	321.63^a^	341.32^a^	264.15^b^	327.45^a^	10.82	0.04	0.08	0.27
Crypt depth, μm	91.01^b^	88.06^b^	138.10^a^	92.46^b^	6.35	0.03	0.02	0.05
V:C	3.53^a^	3.88^a^	1.91^b^	3.54^a^	0.22	0.01	0.01	0.05

*V:C* Villus height:Crypt depth

^1^Mean and total SEM are list in Separate columns, *n* = 8

^2^a, b, c mean values within a row with unlike superscript letters were significantly different *P* < 0.05

^3^*CON*, pigs were fed with a basal diet; *CSL*, pigs were fed with a SL containing diet, 5 g/kg; *ECON*, pigs were fed with a basal diet and challenged by ETEC; *ESL*, pigs were fed with a SL containing diet and challenged by ETEC

**Table 5:** Effect of SL supplementation on mucosal enzyme activity in weaned pigs upon ETEC challenge

Items	Treatments				SEM	*P*-value		
	CON	CSL	ECON	ESL		SL	ETEC	Interaction
Duodenum								
AKP, U/g prot	0.33	0.41	0.29	0.27	0.04	0.68	0.24	0.46
Lactase, U/mg prot	92.95^bc^	107.02^a^	88.61^c^	99.02^ab^	2.21	<0.01	0.06	0.54
Sucrase, U/mg prot	521.99^b^	596.69^a^	498.32^b^	553.51^ab^	14.01	0.01	0.13	0.64
Maltase, U/mg prot	301.48^b^	318.06^a^	286.45^c^	312.27^ab^	4.18	<0.01	0.06	0.37
Jejunum								
AKP, U/g prot	0.21	0.20	0.15	0.24	0.02	0.17	0.66	0.07
Lactase, U/mg prot	86.14	88.44	85.67	85.14	1.12	0.73	0.47	0.58
Sucrase, U/mg prot	497.78^b^	563.07^a^	480.31^b^	556.50^a^	11.06	<0.01	0.43	0.72
Maltase, U/mg prot	290.70^bc^	315.18^a^	286.28^c^	307.49^ab^	4.18	0.01	0.40	0.82
Ileum								
AKP, U/g prot	0.09	0.11	0.07	0.10	0.01	0.18	0.52	0.66
Lactase, U/mg prot	84.65^b^	95.90^a^	76.55^c^	89.47^ab^	2.15	<0.01	0.01	0.75
Sucrase, U/mg prot	513.41^bc^	534.26^a^	506.59^c^	531.65^ab^	4.34	0.00	0.45	0.73
Maltase, U/mg prot	344.98^b^	362.95^a^	342.57^b^	360.23^a^	3.16	0.00	0.59	0.97

*AKP* alkaline phosphatase

^1^Mean and total SEM are list in Separate columns, *n* = 8

^2^a, b, c mean values within a row with unlike superscript letters were significantly different, *P* < 0.05

^3^*CON*, pigs were fed with a basal diet; *CSL*, pigs were fed with a SL containing diet, 5 g/kg; *ECON*, pigs were fed with a basal diet and challenged by ETEC; *ESL*, pigs were fed with a SL containing diet and challenged by ETEC

### Effect of SL supplementation on intestinal microbial populations and metabolites in weaned pigs upon ETEC challenge

As shown in Table 6, ETEC challenge significantly increased the abundance of *E. coli* in the cecum. SL supplementation significantly elevated the abundance of beneficial microbial populations such as the *Lactobacillus*, *Bifidobacterium*, and *Bacillus* in the non-challenged pigs (*P* < 0.05). Moreover, ETEC challenge decreased the concentrations of acetic acid and propanoic acid in the cecum. However, there is a trend that SL supplementation prevented the decreasing concentrations of acetic acid and propanoic acid. SL also elevated the concentration of butyric acid in the non-challenged (*P* < 0.05).

**Table 6:** Effect of SL supplementation on caecal microbial populations and its products in weaned pigs upon ETEC challenge

Items	Treatments				SEM	*P*-value		
	CON	CSL	ECON	ESL		SL	ETEC	Interaction
microbial populations, lg (copies/g)								
Total bacteria	11.81	11.99	11.66	12.03	0.16	0.43	0.88	0.79
*Lactobacillus*	7.89^bc^	9.23^a^	7.37^c^	9.11^a^	0.28	<0.01	0.46	0.65
*Escherichia coli*	7.73^b^	7.43^b^	9.67^a^	8.42^b^	0.27	0.05	<0.01	0.21
*Bifidobacterium*	6.16^bc^	7.00^a^	5.85^c^	6.58^a^	0.15	<0.01	0.16	0.84
*Bacillus*	5.75^b^	6.63^a^	5.71^b^	6.57^a^	0.16	<0.01	0.84	0.96
VFA, μg/g								
Acetic acid	3349.31^a^	3669.06^a^	2504.41^b^	3417.64^a^	133.74	0.01	0.02	0.18
Propanoic acid	1987.11^a^	2103.38^a^	1358.17^b^	2082.78^a^	97.30	0.01	0.05	0.06
Butyric acid	649.89^b^	1150.57^a^	601.12^b^	1097.03^a^	90.68	<0.01	0.71	0.99

*VFA* volatile fatty acids

^1^Mean and total SEM are list in Separate columns, *n* = 8

^2^a, b, c mean values within a row with unlike superscript letters were significantly different, *P* < 0.05

^3^*CON*, pigs were fed with a basal diet; *CSL*, pigs were fed with a SL containing diet, 5 g/kg; *ECON*, pigs were fed with a basal diet and challenged by ETEC; *ESL*, pigs were fed with a SL containing diet and challenged by ETEC

### Effect of SL supplementation on expressions of critical genes involved in intestinal epithelium functions

**Fig. 3:**
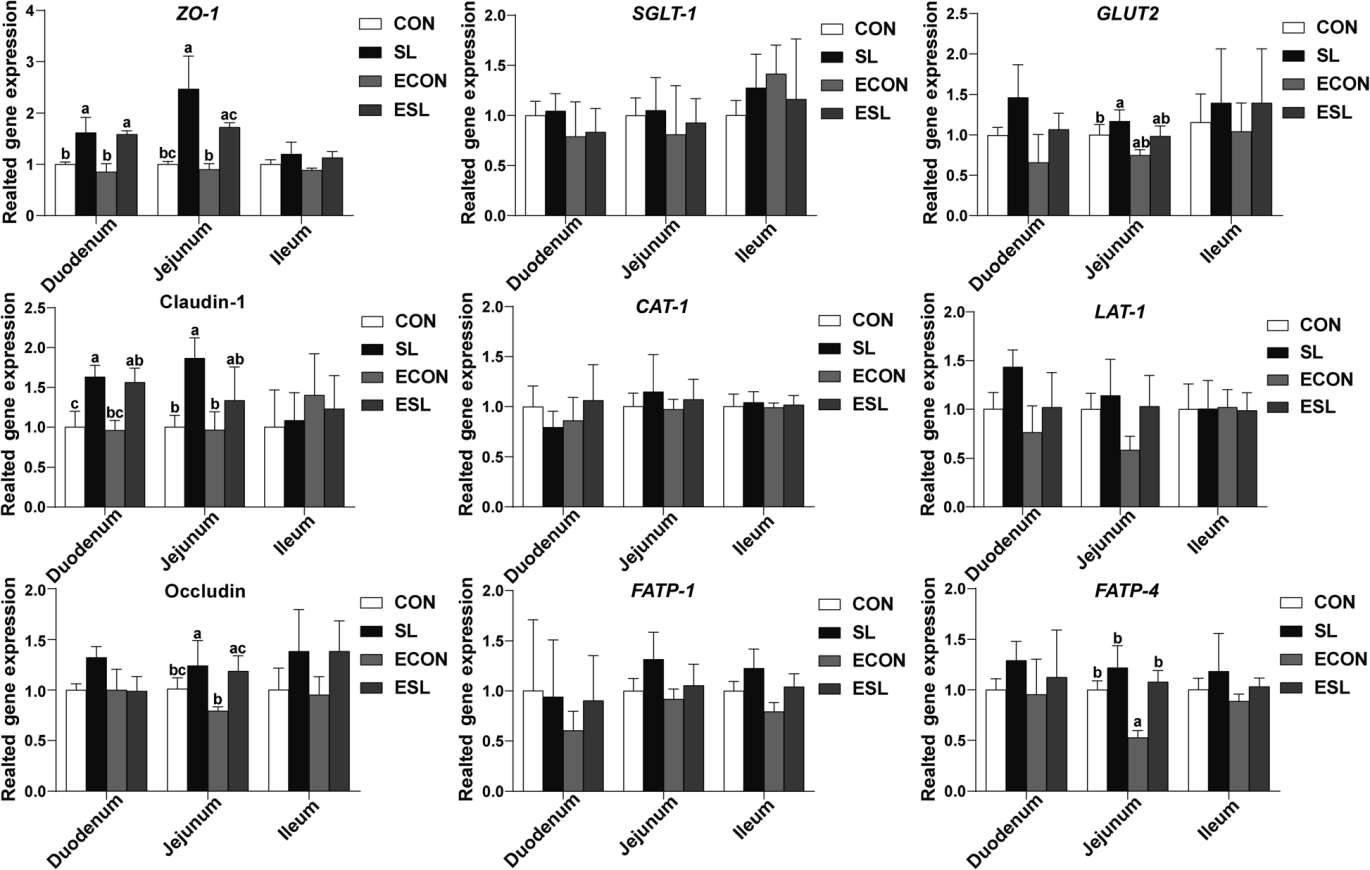
Effect of SL supplementation on mucosal gene expressions in weaned pigs upon ETEC challenge. *ZO-1*, zonula occludens-1. *SGLT-1*, sodium/glucose cotransporter-1; *GLUT-2*, glucose transporter-2; *CAT-1*, cationic amino acid transporter-1; *LAT-1*, L amino acid transporter-1; *FATP*, fatty acid transport proteins; a, b, c mean values within a row with unlike superscript letters were significantly different (*P* < 0.05). CON, pigs were fed with a basal diet; CSL, pigs were fed with a SL containing diet, 5 g/kg; ECON, pigs were fed with a basal diet and challenged by ETEC; ESL, pigs were fed with a SL containing diet and challenged by ETEC

As shown in Fig. 3, SL supplementation significantly elevated the expression levels of *ZO-1* and claudin-1 in the duodenal and jejunal epithelium (*P* < 0.05). SL also elevated the expression level of occludin in the jejunal epithelium (*P* < 0.05). Interestingly, ETEC challenge decreased the expression level of *FATP-4* in the jejunal epithelium. Moreover, SL supplementation also elevated the expression level of jejunal *GLUT-2* in the non-challenged pigs (*P* < 0.05).

## Discussion

It is well known that breastfeeding is the gold standard for infant nutrition. It offers complete nutrients for the newborn as well as many bioactive components that contribute to healthy development of the newborn [26]. Bioactive factors transferred to the infant via breastfeeding including growth factors [27], carbohydrates [28], cells [29], cytokines [30] and immunoglobulins [31]. These bioactive molecules including the MOs were found to shape microbiota composition, modulate gastrointestinal physiology, promote proper development of the immune system, and enhance intestinal barrier function [32]. SL is the major components of the MOs in sows. Previous study indicated that the SL can act as a prebiotic that can promote intestinal development and protect the intestinal epithelium from many harmful pathogens [33]. In the present study, we found that SL not only increased the ADG in the ETEC-challenged pigs, but also increased the digestibility of DM, Ash, and GE in the non-challenged pigs. The result is consistent with previous study [34], and both results suggest a beneficial effect of SL supplementation on the growth performance of the weaned pigs.

Immunoglobulins, also known as antibodies, are glycoprotein molecules produced by plasma cells, which act as a critical part of the immune response by specifically recognizing and binding to particular antigens, such as bacteria or viruses, and aiding in their destruction [35]. In the present study, SL significantly elevated the serum IgA, IgG and IgM concentrations in the ETEC-challenged pigs, indicating an enhanced immunity upon SL supplementation. Pro-inflammatory cytokines such as the TNF-α and IL-1β have been shown to mediate host immune function and produce an immune response quickly after pathogenic microorganisms infection [36]. In the present study, ETEC challenge increased serum concentrations of TNF-α and IL-1β; however, SL significantly decreased the serum concentrations of TNF-α, IL-1β, and IL-6 in the ETEC-challenged pigs. The result is consistent with previous studies, and both indicated an anti-inflammatory property of the MOs [37].

The small intestine is the main place for digestion and absorption, and its integrity determines whether it can function normally. However, ETEC infection can destroy the intestinal structure, and severely impair the intestinal digestion and absorption function [38]. Villi are critical components of the digestive tract, their geometry and V:C ratio provides an indicator of the absorptive capacity of the small intestine [39]. In this study, ETEC challenge decreased the villus height and the V:C in the jejunum and ileum. However, SL significantly improved the intestinal morphology by increasing the villus height and the V:C ratio. This is probably due to the fact that SL reduced the secretion of inflammatory cytokines (such as TNF-α, IL-1β, IL-6) which have been shown to promote apoptosis of the intestinal epithelial cells [40]. Changes of the intestinal morphology is usually accompanied by the digestive enzyme activity in brush border of the intestinal epithelium [41]. In the present study, ETEC challenge decreased the activities of maltase and lactase in the duodenum and ileum. However, SL supplementation significantly elevated their activities both in the non-challenged and ETEC-challenged pigs.

MOs are known to support the growth of beneficial microorganisms in the large intestine, especially the *Bifidobacterium* species, a dominant species in breast-fed infants [42]. As one of the most abundant components of the MOs, SL is highly resistant to hydrolase in the digestive tract and can reach the hindgut in an intact form, where it will be fermented by bacteria to release free sialic acid that can serve as a substrate for bacterial energy metabolism [43, 44]. Recent studies showed that SL can inhibit the adhesion of *E. coli* to Caco-2 cells and increase the abundance of *Bifidobacterium* and *Lactobacilli* in the infants and adults feces [45, 46]. Similar result was obtained in this study and we found that SL significantly increased the abundance of beneficial bacteria such as *Lactobacilli*, *Bacillus*, and *Bifidobacterium* in the non-challenged pigs. Importantly, SL not only decreased the abundance of *Escherichia coli*, but also increased the abundance of *Lactobacilli*, *Bifidobacterium* and *Bacillus* in the ETEC-challenged pigs. Previous study indicated that MOs including the SL are structurally similar to the polysaccharide on the surface of intestinal epithelial cells which can act as an analog of bacterial lectin ligand and block bacterial attachment [45]. Moreover, SL may also change the glycosylation on the surface of the intestinal epithelial cells, and thereby affecting the attachment and colonization of bacteria [47]. Nondigestible oligosaccharides, including SL, can be utilized by microorganisms in large intestine and generate various short-chain fatty acids (SCFA) [48]. SCFA, especially butyric acid, has been shown to play an important role in modulating inflammatory response and intestinal barrier functions [49]. In the present study, SL significantly elevated the concentration of butyric acid in the ETEC-challenged pigs. The result is consistent with previous studies that beneficial microorganisms such as the *Lactobacillus* and *Bifidobacterium* can increase the butyric acid production, which may contribute to reduced generation of pro-inflammatory cytokines such as the TNF-α [50]. Moreover, SL supplementation also significantly elevated the concentration of acetic acid and propionic acid in the ETEC challenged pigs, indicating that SL performs several physiological roles in regulating intestinal permeability, inflammation control and immunological function [51].

Tight junctions are composed of transmembrane barrier proteins (e.g., claudins and occludin), cytoplasmic scaffold proteins (e.g., ZO family), and adhesion molecules, which play a key role in maintaining intestinal permeability [52]. However, various enteric pathogens can alter the distribution of tight junction proteins and impair the permeability of the intestinal epithelium [53]. Previous study indicated that ETEC infection decreased the expression levels of tight junction proteins such as the ZO-1, occluding, and claudins [54]. In the present study, we found that SL supplementation significantly elevated the expression levels of ZO-1 and occludin-1 in the jejunal epithelium upon ETEC challenge. Moreover, SL also elevated the expression levels of GLUT2 and FATP-4 in the jejunal epithelium. GLUT-2 is one of the major transporters for glucose absorption, and the FATP-4 is responsible for long chain fatty acids uptake in enterocytes [55, 56]. These results indicated that dietary SL supplementation is helpful to improve intestinal integrity and epithelial functions in weaned pigs upon ETEC challenge.

In the present study, we found that SL has beneficial effects on intestinal morphology, expression of tight junction proteins and inflammatory cytokines, probiotic colonization and VFA production. Previous studies have shown that probiotics such as *Bifidobacterium* and *Bacillushis* have a protective effect on the intestinal barrier and can downregulate the expression levels of inflammatory cytokines [56–58]. Therefore, we hypothesized that SL could exert a probiotic effect by promoting probiotic colonization, increasing microbial fermentation and breakdown of complex carbohydrates [56], and increasing VFA production.

## Conclusion

Dietary SL supplementation improves the growth performance and alleviates ETEC-induced intestinal injury in the weaned pigs, which was associated with suppressed secretion of inflammatory cytokines and elevated serum immunoglobulins, as well as improved intestinal epithelium functions and microbiota. The beneficial effects of SL on the intestinal integrity and epithelium functions could make it an attractive prebiotic in the use of animal nutrition and feed industry.

## Supplementary Information

**Additional file 1 Table S1**. Sequences of primers for genes and intestinal bacteria.

## Data Availability

The data used to support the findings of this study are available from the corresponding author upon request.

## Abbreviations

ADFI: Average daily feed intake
ADG: Average daily gain
AIA: Acid insoluble ash
AKP: Alkaline phosphatase
CAT-1: Cationic amino acid transporter-1
CON: Pigs were fed with a basal diet
CP: Crude protein
CSL: Pigs were fed with basal diet containing 5 g/kg SL product
Ct: Cycle threshold
DCs: Dendritic cells
DM: Dry matter
ECON: Pigs were fed with a basal diet and challenged by ETEC
EE: Ether extract
ESL: Pigs were fed with basal diet containing 5 g/kg SL product and challenged by ETEC
ETEC: Enterotoxigenic *Escherichia coli*
FATP: Fatty acid transport proteins
Fuc: Fucose
Gal: Galactose
GE: Gross energy
Glc: Glucose
GlcNAc: N-acetylglucosamine
GLM: General linear model
GLUT-2: Glucose transporter-2
IL-1β: Interleukin1-β
IL-6: Interleukin-6
IgA: Immunoglobulins A
IgG: Immunoglobulins G
IgM: Immunoglobulins M
LAT-1: L amino acid transporter-1
LB: Luria-Bertani
MOs: Milk oligosaccharides
Neu5Ac: N-acetylneuraminic acid
PBS: Phosphate buffered saline
PWD: Post weaning diarrhea
SCFA: Short chain fatty acid
SGLT-1: Sodium/glucose cotransporter-1
Sia: Sialic acid
SL: Sialyllactose
TJ: Tight junctions
TNF-α: Tumor necrosis factor-α
V:C: Villus height : crypt depth
VFA: Volatile fatty acids
ZO-1: Zonula occludens-1
