# Supplementary material for: Effect of sialyllactose on growth performance and intestinal epithelium functions in weaned pigs challenged by enterotoxigenic Escherichia Coli
Source: J Anim Sci Biotechnol. 2022 Mar 3;13:30. doi: 10.1186/s40104-022-00673-8 (PMC8892705; doi:10.1186/s40104-022-00673-8)
Supplement: Supplementary file 1 — Additional file 1 Table S1. Sequences of primers for genes and intestinal bacteria. [file 40104_2022_673_MOESM1_ESM.docx]

Table S1. Sequences of primers for genes and intestinal bacteria

| Gene | Primer sequence (5’ to 3’) | Annealing temperature, ℃ | Product size, bp |
| --- | --- | --- | --- |
| β-Actin | F: TGGAACGGTGAAGGTGACAGC | 60 | 177 |
|  | R: GCTTTTGGGAAGGCAGGGACT |  |  |
| *SGLT-1* | F: CCACTTTCCCTATAAAACCTCAC | 60 | 151 |
|  | R: CTCCATCAAACTTCCATCCTCAG |  |  |
| *GLUT-2* | F: CCTGCTTGGTCTATCTGCTGTG | 60 | 156 |
|  | R: TTGATGCTTCTTCCCTTTCTTT |  |  |
| *CAT-1* | F: TGCCCATACTTCCCGTCC | 60 | 192 |
|  | R: GGTCCAGGTTACCGTCAG |  |  |
| *LAT-1* | F: GCCCATTGTCACCATCATC | 60 | 216 |
|  | R: GAGCCCACAAAGAAAAGC |  |  |
| *FATP-1* | F: GGAGTAGAGGGCAAAGCAGG | 60 | 208 |
|  | R: AGGTCTGGCGTGGGTCAAAG |  |  |
| *FATP-4* | F: TTCATCAAGACGGTCAGGCG | 60 | 133 |
|  | R: AGACGGTGGCAGCGAATAAG |  |  |
| *ZO-1* | F: CAGCCCCCGTACATGGAGA | 60 | 114 |
|  | R: GCGCAGACGGTGTTCATAGTT |  |  |
| Occludin | F: CTACTCGTCCAACGGGAAAG | 60 | 158 |
|  | R: ACGCCTCCAAGTTACCACTG |  |  |
| Claudin-1 | F: GCCACAGCAAGGTATGGTAAC | 60 | 140 |
|  | R: AGTAGGGCACCTCCCAGAAG |  |  |
| Total bacteria | F: ACTCCTACGGGAGGCAGCAG | 60 | 200 |
|  | R: ATTACCGCGGCTGCTGG |  |  |
| *Lactobacillus* | F: GAGGCAGCAGTAGGGAATCTTC | 60 | 126 |
|  | R: CAACAGTTACTCTGACACCCGTTCTTC |  |  |
|  | P: AAGAAGGGTTTCGGCTCGTAAAACTCTGTT |  |  |
| *Escherichia coli* | F: CATGCCGCGTGTATGAAGAA | 60 | 96 |
|  | R: CGGGTAACGTCAATGAGCAAA |  |  |
|  | P: AGGTATTAACTTTACTCCCTTCCTC |  |  |
| *Bifidobacterium* | F: CGCGTCCGGTGTGAAAG | 60 | 121 |
|  | R: CTTCCCGATATCTACACATTCCA |  |  |
|  | P: ATTCCACCGTTACACCGGGAA |  |  |
| *Bacillus* | F: GCAACGAGCGCAACCCTTGA | 60 | 92 |
|  | R: TCATCCCCACCTTCCTCCGGT |  |  |
|  | P: CGGTTTGTCACCGGCAGTCACCT |  |  |

*SGLT-1*, sodium/glucose cotransporter 1; *GLUT-2*, glucose transporter 2; *CAT-1*, cationic AA transporter 1; *LAT-1*, L amino acid transporter 1; *FATP-1*, fatty acid transport protein 1; *FATP-4*, fatty acid transport protein 4; *ZO-1*, zonula occludens 1.
